# Inhospital Mortality in Patients with Type 2 Diabetes Mellitus: A Prospective Cohort Study in Lima, Peru 

**DOI:** 10.1155/2016/7287215

**Published:** 2015-12-16

**Authors:** Henry Zelada, Antonio Bernabe-Ortiz, Helard Manrique

**Affiliations:** ^1^Internal Medicine Training Program, Louis A. Weiss Memorial Hospital, 4646 N. Marine Drive, Chicago, IL 60640, USA; ^2^School of Public Health and Administration, Peruvian University Cayetano Heredia, Lima, Peru; ^3^School of Medicine, Peruvian University Cayetano Heredia, Lima, Peru; ^4^Center of Excellence in Chronic Diseases (CRONICAS), Peruvian University Cayetano Heredia, Lima, Peru; ^5^School of Medicine, Peruvian University of Applied Sciences, Lima, Peru; ^6^Arzobispo Loayza National Hospital, Lima, Peru

## Abstract

*Objective*. To estimate cause of death and to identify factors associated with risk of inhospital mortality among patients with T2D.* Methods*. Prospective cohort study performed in a referral public hospital in Lima, Peru. The outcome was time until event, elapsed from hospital admission to discharge or death, and the exposure was the cause of hospital admission. Cox regression was used to evaluate associations of interest reporting Hazard Ratios (HR) and 95% confidence intervals.* Results*. 499 patients were enrolled. Main causes of death were exacerbation of chronic renal failure (38.1%), respiratory infections (35.7%), and stroke (16.7%). During hospital stay, 42 (8.4%) patients died. In multivariable models, respiratory infections (HR = 6.55, *p* < 0.001), stroke (HR = 7.05, *p* = 0.003), and acute renal failure (HR = 16.9, *p* = 0.001) increased the risk of death. In addition, having 2+ (HR = 7.75, *p* < 0.001) and 3+ (HR = 21.1, *p* < 0.001) conditions increased the risk of dying.* Conclusion*. Respiratory infections, stroke, and acute renal disease increased the risk of inhospital mortality among hospitalized patients with T2D. Infections are not the only cause of inhospital mortality. Certain causes of hospitalization require standardized and aggressive management to decrease mortality.

## 1. Introduction

Worldwide, an estimated 382 million people live with type 2 diabetes (T2D), causing at least US 548 billion dollars in health care expenditures [1], and 80% of these people were living in low- and middle-income countries (LMIC) [2]. Besides, approximately 5.1 million individuals die because of T2D and nearly half of these deaths occur in people under the age of 60 [1].

There is scarce information regarding mortality rates and causes of death in patients with T2D in LMIC. For many years, infections have been considered the main cause of mortality in LMIC, despite the fact that vascular diseases, especially stroke and myocardial infarction, are the underlying causes in high-income countries [3, 4]. Due to the relatively fast nutritional and epidemiological transition LMIC are going through, it is necessary to determine potential causes of death among T2D patients in resource-constrained settings to implement appropriate strategies.

Information regarding inhospital mortality rates as well as clinical conditions increasing the risk of dying among T2D patients is very limited in resource-constrained settings. As a result, there is a lack of preventive policies in high-risk T2D cases that might reduce and prevent inhospital mortality. For example, a previous study, conducted in Peru in 1996, reported that infections were the main cause of morbidity and mortality in a public referral hospital [5]; however, current circumstances might have changed.

As a result, the aim of this study was to identify potential clinical factors that might increase the risk of inhospital mortality among T2D patients. In addition, mortality rate and main causes of hospital admission and death were also determined. Results of this study can be helpful to implement public health strategies and update hospital management guidelines in order to prevent and reduce inhospital mortality among these kind of patients.

## 2. Methods

### 2.1. Study Design and Setting

A prospective cohort study was conducted in the Internal Medicine Hospitalization Unit at the Hospital Nacional Arzobispo Loayza (HNAL) located in Lima, Peru. HNAL is a third-level public hospital located in the center of Lima, with more than 2500 hospital admissions monthly in the different available services and more than 30,000 outpatients attending every month [6].

### 2.2. Selection of Participants

Patients with T2D diagnosis, confirmed by an internal medicine physician or endocrinologist in the emergency or ambulatory room, aged 18 or more and admitted to the Internal Medicine Service of the HNAL during the year 2012 were enrolled in the study. In case of multiple hospitalizations during the year, only the first hospital admission was included in the analyses. Pregnant women and patients admitted for diagnostic or therapeutic procedures such as kidney biopsy, peritoneal dialysis catheter placement, or corticosteroid pulse were excluded.

### 2.3. Variables Definition

The outcome of interest was time until event defined as the time, in days, elapsed from admission until the patient was discharged by medical indication or voluntary decision (censored) or died during hospitalization.

The exposure of interest was the cause of hospitalization defined as the diagnosis at the moment of hospital admission. Only one physician, one of the authors of this paper, was responsible for verifying and confirming the cause of hospitalization during follow-up. Causes of hospitalization were subdivided into the most common reasons previously reported including infections (urinary, respiratory, subcutaneous tissue, or diabetic foot), metabolic disorders (hypoglycemia, diabetic ketoacidosis, and hyperosmolar state), stroke, and acute and chronic renal failure (exacerbation). Definitions of the variables used are detailed in Table 1. In addition, causes of hospitalization were also grouped by addition and then assessed as an independent exposure as T2D patients might present different morbidities at the same time.

Clinical variables, especially related to T2D, were also considered including time of disease, in years, split in four categories (<5, 5–9, 10–14, and 15+), type of hospital admission (outpatient service or emergency), and medicines received prior to hospital admission (yes/no). Besides, glycated hemoglobin (HbA1c) level was categorized as noncontrolled (≥7%) and controlled (<7%), according to international guidelines [7]. Finally, sociodemographic variables were also evaluated as potential confounders, including gender, age in years (<50, 50–59, 60–69, or ≥70), region of birth (coast, highlands, or jungle), and education level (<7 years, 7–11 years, or ≥12 years).

### 2.4. Procedures

Previous informed consent, third-year residents of Endocrinology evaluated the patients that fulfill the inclusion criteria for the study. Residents were trained before starting fieldwork activities to appropriately applied questionnaires and complete data collection templates. Sociodemographic, clinical, and laboratory data was recorded at the moment of hospital admission. Patients were then followed up for diagnosis confirmation as well as recording the outcome of interest (discharge or death).

### 2.5. Sample Size and Power

Using Power and Sample Size software (PASS 2008, NCSS, UTAH, US), with information from 499 participants and assuming a level of significance of 5% and a mortality rate of 0.57 per 100 persons-day of follow-up, we have a power of 80% to detect a Hazard Ratio of 3 or more.

### 2.6. Data Analysis

STATA 13 for Windows (STATA Corporation, College Station, Texas, US) was used for statistical analysis. Initially, the study population was described using mean and standard deviation for numerical variables and proportions for categorical variables. Association between inhospital mortality rate and sociodemographic, clinical variables and causes of hospitalization were assessed using Log-rank test. After that, Cox regression crude and adjusted models were fitted to evaluate associations between causes of hospitalization (individual and grouped) and our outcome of interest, reporting Hazard Ratios (HR) and their respective 95% confidence intervals (95% CI).

Because a large proportion of patients (40.2%) did not have HbA1c results and 11.8% did not have time of disease information, with both considered important confounders in the models, the associations of interest were also evaluated using multiple imputation techniques, based on data collected. As recommended in previous studies [8–10] and because of being feasible, 20 imputations were made to reduce sample errors. Despite not being considered a good practice [11], association was also evaluated using Cox regression models with an extra “missing value” category in HbA1c and time of disease variables.

### 2.7. Ethics

The study was reviewed and approved by the Institutional Ethical Committee of Universidad Peruana Cayetano Heredia, Lima, Peru. Oral informed consent was requested from participants before fieldwork activities. Data collection templates contained alphanumeric codes to avoid personal identifiers.

## 3. Results

### 3.1. Characteristics of the Study Population

A total of 499 patients were enrolled during the study, 63.6% female, mean age 61.6 (SD: 13.8) years. Of note, 33.6% of patients reported 15 or more years of disease, only 343 (68.6%) were receiving treatment before hospitalization (20.5% with insulin and 62.4% with metformin), and only 71/299 (23.8%) met recommended glycemic goals (HbA1c < 7%). Among infections, the main causes of hospital admission were urinary infections (23.0%), followed by diabetic foot (22.4%). Among noncommunicable diseases, the main cause of hospitalization was exacerbation of chronic renalfailure (18.8%) and stroke (5.6%). Of note, only 4 cases (0.8%) were admitted with diagnosis of acute myocardial infarction.

### 3.2. Death during Hospitalization and Causes

During the study, 42 (8.4%) patients died with a median survival time of 7 days (interquartile range: 2–17). Detailed characteristics of the study population according to the outcome of interest are shown in Table 2. Overall mortality rate per 100 persons-day of follow-up was 0.57 (95% CI: 0.42–0.78) and varied from 0.21 among those with diabetic foot to 10.7 among those with acute renal disease (Table 3). None of the demographic and T2D-related variables were associated with mortality. The main causes of death were exacerbation of the chronic renal failure (38.1%), followed by respiratory infections (35.7%) and stroke (16.7%).

### 3.3. Reasons of Hospitalization and Inhospital Mortality: Multivariable Models

Respiratory infections (*p* = 0.001), hyperosmolar state (*p* = 0.002), stroke (*p* = 0.003), and acute renal failure (*p* = 0.001) increased the risk of inhospital mortality after adjusting for gender, age, place of origin, education level, time of disease, hospital admission, treatment, and glycated hemoglobin levels. On the other hand, only patients admitted with diagnosis of diabetic foot had lower probability of dying during follow-up (*p* = 0.02) in the adjusted model (Table 3).

Results were consistent when models were fitted using multiple imputation techniques; however, hyperosmolar state was no longer significant (*p* = 0.51) and chronic renal failure (exacerbation) became a risk factor (*p* = 0.01) in multivariable models. Similarly, when the analysis was performed by adding an extra “missing value” category in HbA1c and time of disease variables, the results were similar to multiple imputation models (see online information, Table E-1, in the Supplementary Material available online at http://dx.doi.org/10.1155/2016/7287215).

Finally, when the sum of causes of admission was assessed, an increase in the number of conditions (comorbidities) was associated with greater risk of mortality. Although results of imputed models were consistent, values of HR were markedly attenuated (Table 4).

## 4. Discussion

### 4.1. Main Findings

In the present study, respiratory infections, stroke, and acute renal failure were positively associated with dying during hospitalization after controlling for several potential cofounders. Conversely, a hospital admission with the diagnosis of diabetic foot was negatively associated with mortality. In addition, an increasing number of morbidities were associated with greater risk of death.

### 4.2. Inhospital Mortality and Associated Risk Factors

There are few prospective studies evaluating diagnosis at the moment of hospital admission as potential risk factors for inhospital mortality among T2D patients. One of the most recent studies, conducted in UK [12], identified that hospital-admitted T2D patients had 6.4% greater risk of dying over two years than what would be expected compared to similar patients without diabetes. In addition, according to that study, the risk of death varied according to reason of admission: cardiac disease or urinary tract disease showed the highest number of additional deaths. However, most of the inhospital mortality studies are retrospective in nature. A 7-year cohort study using the General Practice Research Database in UK reported an all-cause mortality estimate of 9.8% among T2D patients and 3.0% of cardiovascular deaths [13]. Additionally, a previous study showed that mortality patterns changed according to race; thus, blacks were at lower risk of dying from ischemic heart disease or respiratory disorders than whites but at higher risk due to renal failure, heart failure, and cancer [14]. On the other hand, two studies conducted in India reported divergent results: one of them showed a mortality rate of 7.1% and infections were the leading cause of death, followed by chronic renal failure, coronary artery disease, and cerebrovascular disease [15], whereas a recent one reported vascular disease as the leading cause of death [4].

Two previous studies performed several years ago in Peru deserve attention. One of them reported that more than half (52%) of the hospital admissions in T2D patients were due to infections, mainly focused on soft tissues and urinary and respiratory tract, followed by chronic renal failure (24%) and heart failure (7%) [16]. The other study reported that 7.6% of patients admitted to hospitalization died and infections were the leading cause of inhospital mortality [5]. Comparing with our results, inhospital mortality was almost the same, but causes of death might have changed over time.

Although infections still increase inhospital mortality among T2D patients, other causes, especially stroke, and acute renal failure, are also potential risk factors for inhospital mortality. As countries are at different stages of nutrition and epidemiological transition, it is possible that causes of death can vary according to the setting evaluated. Then, there is a need to understand characteristics associated with the risk of death in each country and even within it. For example, about 70% of patients must pay to receive health care in public hospitals, even when most of the T2D patients are covered by the national health insurance (SIS in Spanish). This might, then, imply a new challenge in the health care system as appropriate strategies are needed in order to prevent and reduce inhospital mortality.

The negative association between diabetic foot infection and mortality merits some comments. As diabetic foot infection was one of the main causes of hospital admission in Peru [16], standardized clinical guidelines [17] have been adapted to adequately treat this complication in our context; as a result, cases with diabetic foot are treated aggressively. Moreover, amputation can help to reduce or delay death in this group [18]. A previous study from Iran reported that 5.6% of hospital-admitted T2D cases with diabetic foot infection died due mainly to sepsis [19]. In our study, 4.5% of cases with diabetic foot infection died during hospitalization, most of them (72.1%) with grade 3, 4, or 5 in the Wagner classification of diabetic foot [20].

### 4.3. Additional Findings

Only a quarter of hospital-admitted patients met recommended HbA1c levels, carrying out particular challenges especially in resource-constrained settings including rate of complications, number of hospitalizations, and out-of-pocket payments to cover health care [21]. Adequate glycemic control reduces the risk of developing complications such as stroke, cardiovascular events, amputation, and chronic renal disease [22]. However, several studies in different settings have reported suboptimal glycemic control rates among T2D patients [23–25]. Moreover, a decrease in adequate glycemic control rates has been also observed during past years in some locations [26]. In addition, two-thirds of patients reported not receiving antidiabetic treatment before hospitalization, which together with low glycemic control could explain the occurrence of complications and the need for hospitalization. On the other hand, during hospitalization, 66% of the patients received any form of insulin therapy, whereas only 16% received oral antidiabetic treatment (data not shown).

### 4.4. Limitations and Strengths

Strength of this study included its prospective nature, the standardized assessment conducted by one health care staff to verify and confirm the diagnosis and the reason of hospitalization, and the use of one of the main referral hospitals in Lima to perform the study. This study, however, has some limitations. First, the high proportion of missing values in the case of HbA1c and time of disease variables could have affected our results. Although other studies have had the same problem [27, 28], our results were consistent using different techniques, including multiple imputation models. Second, some variables used in previous studies [4, 29] were not considered in our regression models, including socioeconomic status, race, the number of previous hospital admissions, history of cancer, fasting glucose assessment, depression as well as other mental illnesses, and potassium and sodium levels [3, 4, 12, 13]. Finally, some selection bias may arise due to the selection of a referral hospital instead of primary care facilities or T2D patients from the general population. Therefore, our results, although important, cannot be inferable to different health and social realities. However, we expect that results can be applicable to similar contexts.

## 5. Conclusions

Respiratory infections, stroke, and acute renal disease, as diagnosis of hospital admission, increased the risk of in-hospital mortality among T2D patients, whereas the presence of diabetic foot was a protective factor. In addition, an increasing number of conditions were also associated with greater risk of mortality. Results of this study confirm that infections are not the only potential cause of inhospital mortality in a developing country and certain causes of hospital admission require a more aggressive and standardized management in order to decrease mortality among T2D patients.

## Supplementary Material

Table E1: Multivariable Cox regression model including glycated hemoglobin missing values as an additional category.
In order to evaluate the impact of the glycated hemoglobin missing of values over our regression models, we decided to compare them to a new model that includes missing glycated hemoglobin as an additional variable. No major changes in HR resulted; respiratory infections, stroke, acute renal failure and chronic renal failure continued being high risk factors for inpatient mortality and diabetic foot continued as a protective factor for inpatient mortality in patients with T2D.

## Figures and Tables

**Table 1:** Definition of causes of hospitalization assessed in the study.

Infections	
Respiratory	Respiratory symptoms (cough or tachypnea) plus a chest X-ray with changes suggestive of viral or bacterial respiratory infection.
Urinary	Urine sample with ≥10 leukocytes/*µ*L [30], temperature > 38.0°C, and not being able to orally tolerate fluids/food.
Gastrointestinal	Diarrhea < 7 days, vomiting, and dehydration.
Subcutaneous tissue (SCT)	Cellulitis or necrotizing fasciitis in any part of the body except feet.
Diabetic foot	Ulceration, infection, and/or gangrene of foot associated with diabetic neuropathy and different grades of peripheral artery disease [21].

Metabolic disorders	
Hypoglycemia	Glucose ≤70 mg/dL (3.9 mmol/L) [21].
Diabetic ketoacidosis	Glucose >250 mg/dL, pH <7.3, and bicarbonate <18 mEq/d [21].
Hyperosmolar state	Glucose >600 mg/dL, pH arterial: >7.30, bicarbonate: >18 mEq/L, anion GAP: variable, mental status: drowsy/coma, few kenotic bodies in the urine and blood, and plasmatic osmolality > 320 mOsm/kg [21].

Vascular	
Stroke	Fast development of clinic signs of changes in the cerebral function or global, with symptoms that persist within 24 hours or more, with no other evidence of vascular origin [21].

Renal	
Acute renal failure	Sudden increase (within 48 hours) of creatinine (Cr) ≥0.3 mg/dL (26.4 micromol/L) of basal or a percentage of increment of Cr of ≥50%; or oliguria of <0.5 mL/kg/hour by more than six hours [31].
Chronic renal failure	Presence of renal damage (urinary albumin excretion ≥30 mg/day) or decrease of the renal function (GFR <60 mL/min/1.73 m^2^) by three or more months, independent of the cause [32] documented as past medical history plus acute renal failure at the moment of admission (exacerbation).

**Table 2:** Sociodemographic factors associated with death during hospitalization.

	Time to event (mean, in days)	Dead/total (*n* = 42)	*p* value^*∗*^
Gender			
Female	9.9	30/314 (9.5%)	0.15
Male	17.8	12/180 (6.7%)	
Age (years)			
<50 years	11.3	3/91 (3.3%)	0.12
50–59 years	10.6	9/132 (6.8%)	
60–69 years	11.7	14/125 (11.2%)	
70+ years	13.5	16/149 (10.7%)	
Place of origin			
Coast	11.8	36/418 (8.6%)	0.90
Highlands	13.0	4/60 (6.7%)	
Jungle	16.0	2/19 (10.5%)	
Education level			
<7 years	8.6	21/236 (8.9%)	0.52
7–11 years	21.1	13/204 (6.4%)	
12+ years	5.0	5/51 (9.8%)	
Time of disease (years)			
<5 years	9.8	13/131 (9.9%)	0.97
5–9 years	17.7	6/77 (7.8%)	
10–14 years	5.0	6/85 (7.1%)	
15+ years	13.7	14/148 (9.5%)	
Hospital admission			
Outpatient	12.3	39/465 (8.4%)	0.73
Emergency	10.0	3/33 (9.1%)	
Receiving treatment before admission			
No	10.5	15/156 (9.6%)	0.35
Yes	13.0	27/343 (7.9%)	
Glycemic control (HbA1c)			
Controlled (<7%)	11.1	8/71 (11.3%)	0.14
Uncontrolled (≥7%)	10.7	13/228 (5.7%)	

^*∗*^Log-rank test was used to calculate *p* values.

**Table 3:** Factors associated with mortality during hospital admission: crude and adjusted models using Cox regression.

		Dead/total	Mortality	Crude model	Adjusted model^*∗*^	Imputed model^*∗∗*^
		(*n* = 42)	Rate (95% CI)	HR (95% CI)	HR (95% CI)	HR (95% CI)
Urinary infection	No	35/385 (9.1%)	0.59 (0.42–0.83)	1 (Reference)	1 (Reference)	1 (Reference)
	Yes	7/114 (6.1%)	0.48 (0.23–1.01)	0.77 (0.34–1.75)	1.04 (0.27–3.98)	0.70 (0.28–1.72)
Respiratory infection	No	27/438 (6.2%)	0.41 (0.28–0.61)	1 (Reference)	1 (Reference)	1 (Reference)
	Yes	15/61 (24.6%)	1.68 (1.01–2.78)	**4.26 (2.25–8.10)**	**6.55 (2.09–20.50)**	**5.62 (2.53–12.50)**
Gastrointestinal infection	No	38/461 (8.2%)	0.55 (0.40–0.76)	1 (Reference)	1 (Reference)	1 (Reference)
	Yes	4/38 (10.5%)	0.82 (0.31–2.19)	1.49 (0.53–4.19)	1.04 (0.12–8.79)	1.91 (0.65–5.64)
Subcutaneous infection	No	36/459 (7.8%)	0.53 (0.38–0.74)	1 (Reference)	1 (Reference)	1 (Reference)
	Yes	6/39 (15.4%)	1.14 (0.51–2.53)	2.18 (0.91–5.19)	1.71 (0.33–8.79)	1.36 (0.45–4.09)
Diabetic foot	No	36/387 (9.3%)	0.73 (0.52–1.02)	1 (Reference)	1 (Reference)	1 (Reference)
	Yes	5/111 (4.5%)	0.21 (0.09–0.51)	**0.30 (0.11–0.77)**	**0.13 (0.02–0.74)**	**0.28 (0.10–0.76)**
Hypoglycemia	No	39/449 (8.7%)	0.57 (0.41–0.78)	1 (Reference)	1 (Reference)	1 (Reference)
	Yes	3/50 (6.0%)	0.68 (0.22–2.10)	1.07 (0.33–3.50)	0.80 (0.09–6.88)	0.73 (0.17–3.21)
Diabetic ketoacidosis	No	40/462 (8.7%)	0.58 (0.43–0.80)	1 (Reference)	1 (Reference)	1 (Reference)
	Yes	2/36 (5.6%)	0.44 (0.11–1.74)	0.72 (0.17–2.97)	4.77 (0.49–46.71)	2.07 (0.46–9.37)
Hyperosmolar state	No	40/482 (8.3%)	0.56 (0.41–0.76)	1 (Reference)	1 (Reference)	1 (Reference)
	Yes	2/17 (11.8%)	1.07 (0.27–4.28)	1.79 (0.43–7.45)	**17.69 (2.88–108.8)**	1.66 (0.37–7.44)
Stroke	No	35/471 (7.4%)	0.50 (0.36–0.70)	1 (Reference)	1 (Reference)	1 (Reference)
	Yes	7/28 (25.0%)	1.70 (0.81–3.56)	**3.38 (1.49–7.68)**	**7.05 (1.91–26.07)**	**3.52 (1.46–8.45)**
Acute renal disease	No	39/493 (7.9%)	0.53 (0.39–0.73)	1 (Reference)	1 (Reference)	1 (Reference)
	Yes	3/5 (60.0%)	10.7 (3.46–33.2)	**11.78 (3.60–38.53)**	**16.89 (3.10–91.96)**	**13.73 (3.81–49.47)**
Chronic renal disease	No	26/405 (6.4%)	0.43 (0.29–0.63)	1 (Reference)	1 (Reference)	1 (Reference)
	Yes	16/94 (17.0%)	1.24 (0.76–2.02)	**2.91 (1.55–5.49)**	2.69 (0.77–9.45)	**2.73 (1.26–5.93)**

Mortality rates were calculated per 100 persons-day of follow-up.

^*∗*^The model was adjusted for gender, age, place of origin, education level, time of disease, hospital admission, treatment, and glycemic control.

^*∗∗*^The imputed model was adjusted for the same variables listed above; missing values of glycemic control and time of disease were imputed.

**Table 4:** Cumulative effect of comorbidities and its association with death: crude and adjusted models using Cox regression.

	Alive	Dead	Crude model	Adjusted model^*∗*^	Imputed model^*∗∗*^
	(*n* = 457)	(*n* = 42)	HR (95% CI)	HR (95% CI)	HR (95% CI)
Number of morbidities					
One	331 (94.8%)	18 (5.2%)	1 (Reference)	1 (Reference)	1 (Reference)
Two	105 (87.5%)	15 (12.5%)	**2.76 (1.36–5.60)**	**7.75 (1.62–37.01)**	**2.39 (1.07–5.32)**
Three or more	18 (69.2%)	8 (30.8%)	**7.19 (3.07–16.82)**	**21.12 (4.43–100.86)**	**9.70 (3.56–26.40)**

The number of morbidities was calculated by adding proposed causes of hospitalization.

^*∗*^The model was adjusted for gender, age, place of origin, education level, time of disease, hospital admission, treatment, and glycemic control.

^*∗∗*^The imputed model was adjusted for the same variables listed above; missing values of glycemic control and time of disease were imputed.

## Novelty Statement

There are not enough prospective studies assessing risk factors of inhospital mortality in T2D patients, especially in low- and middle-income countries (LMIC), such as Peru. Different regression models were generated to evaluate the association between reason of hospitalization and inhospital mortality. We found that infections are not the only cause of inhospital mortality, as the literature used to report, but also stroke and acute renal disease increased the risk of inhospital mortality in a transitioning setting.
