# Supplementary material for: Inhospital Mortality in Patients with Type 2 Diabetes Mellitus: A Prospective Cohort Study in Lima, Peru
Source: J Diabetes Res. 2015 Dec 16;2016:7287215. doi: 10.1155/2016/7287215 (PMC4695674; doi:10.1155/2016/7287215)
Supplement: Supplementary file 1 — Table E1: Multivariable Cox regression model including glycated hemoglobin missing values as an additional category. In order to evaluate the impact of the glycated hemoglobin missing of values over our regression models, we decided to compare them to a new model that includes missing glycated hemoglobin as an additional variable. No major changes in HR resulted; respiratory infections, stroke, acute renal failure and chronic renal failure continued being high risk factors for inpatient mortality and diabetic foot continued as a protective factor for inpatient mortality in patients with T2D. [file 7287215.f1.pdf]

Table E1: Multivariable Cox regression model including glycated hemoglobin missing values as an additional category

	Adjusted model HR (95%CI)	p-value
Urinary infection		
Yes	0.78 (0.32 – 1.92)	0.59
Respiratory infection		
Yes	**4.93 (2.40 – 10.11)**	<0.001
Gastrointestinal infection		
Mean (SD)	2.02 (0.69 – 5.94)	0.20
Subcutaneous infection		
Yes	1.81 (0.61 – 5.37)	0.29
Diabetic foot		
Yes	**0.27 (0.10 – 0.74)**	0.01
Hypoglycemia		
Yes	0.71 (0.16 – 3.08)	0.65
Diabetic ketoacidosis		
Yes	3.31 (0.72 – 15.19)	0.12
Hyperosmolar state		
Yes	3.74 (0.78 – 17.87)	0.10
Stroke		
Yes	**3.96 (1.63 – 9.64)**	0.002
Acute kidney disease		
Yes	**16.06 (4.29 – 60.17)**	<0.001
Chronic kidney disease (exacerbation)		
Yes	**2.48 (1.18 – 5.20)**	0.02

* The adjusted model was adjusted for gender, age, place of origin, education level, time of disease, hospital admission, treatment, and glycated hemoglobin. Glycated hemoglobin was included in the model as a three-category variable (uncontrolled, controlled, and missing values)
